# Assessing Basal and Acute Autophagic Responses in the Adult *Drosophila* Nervous System: The Impact of Gender, Genetics and Diet on Endogenous Pathway Profiles

**DOI:** 10.1371/journal.pone.0164239

**Published:** 2016-10-06

**Authors:** Eric P. Ratliff, Roxanne W. Kotzebue, Brandon Molina, Ruth E. Mauntz, Arysa Gonzalez, Ayeh Barekat, Nadja El-Mecharrafie, Shannon Garza, Michael A. Gurney, Madhulika Achal, Phyllis-Jean Linton, Greg L. Harris, Kim D. Finley

**Affiliations:** 1 Donald P. Shiley BioScience Center, San Diego State University, San Diego, California, United States of America; 2 Department of Biology, San Diego State University, San Diego, California, United States of America; Children's Hospital of Pittsburgh, University of Pittsburgh Medical Center, UNITED STATES

## Abstract

The autophagy pathway is critical for the long-term homeostasis of cells and adult organisms and is often activated during periods of stress. Reduced pathway efficacy plays a central role in several progressive neurological disorders that are associated with the accumulation of cytotoxic peptides and protein aggregates. Previous studies have shown that genetic and transgenic alterations to the autophagy pathway impacts longevity and neural aggregate profiles of adult *Drosophila*. In this study, we have identified methods to measure the acute *in vivo* induction of the autophagy pathway in the adult fly CNS. Our findings indicate that the genotype, age, and gender of adult flies can influence pathway responses. Further, we demonstrate that middle-aged male flies exposed to intermittent fasting (**IF**) had improved neuronal autophagic profiles. IF-treated flies also had lower neural aggregate profiles, maintained more youthful behaviors and longer lifespans, when compared to *ad libitum* controls. In summary, we present methodology to detect dynamic *in vivo* changes that occur to the autophagic profiles in the adult Drosophila CNS and that a novel IF-treatment protocol improves pathway response in the aging nervous system.

## Introduction

A complex mixture of genetic and environmental factors can influence the aging process in the nervous system, which in turn impacts the accumulation of protein aggregates and the maintenance of complex behaviors [1–3]. Age-dependent neural defects have been well characterized and have largely focused on the damage caused by reactive oxygen species (ROS), as well as the production of cytotoxic protein aggregates that disrupt neuronal function [2, 4–6]. The accumulation of neural aggregates, caused by alterations in protein homeostasis, has also been closely associated with several degenerative disorders and behavioral defects in people [1, 7, 8]. This includes neurological diseases that can be classified as genetic (familial) or sporadic in origin [7, 9, 10]. Genetic mutations that influence protein folding (*i*.*e*. alpha-synuclein) [7], aggregation tendencies (*i*.*e*. Poly-Q) [11, 12], or protein processing, resulting in the generation of cytotoxic peptides (*i*.*e*. amyloid-beta), have highlighted a genetic basis for some neurological diseases [13–16]. However, the most common progressive neurodegenerative disorders have not been linked to specific genetic defects (sporadic) and are often associated with progressive age-dependent changes to metabolic signaling (insulin/TOR) or cellular clearance pathways [9, 13, 14, 17–19].

One such clearance pathway is macroautophagy (autophagy), which is a highly conserved sequestration and vesicle transport system that intersects with the lysosomal and endosomal pathways [14, 18, 20–22]. Autophagy occurs at basal levels in most cells and tissues and requires the *de novo* formation of autophagosomes [21]. However, depending on changing physiological conditions, which influence multiple upstream signaling pathways, autophagic activity can be dramatically altered. This in turn impacts pathway flux and the subsequent degradation of material by the lysosome [23, 24]. While direct links between autophagic defects and neurological disorders are still being examined, mutagenesis studies have shown that loss of autophagic function accelerates protein aggregate accumulation and the decline of the nervous system [5, 18, 25]. In addition, a common feature of many human neural aggregates is the presence of the intracellular scaffolding protein p62 (SQSTM1) and ubiquitinated proteins [18, 26–28]. The p62 protein and its *Drosophila* homolog, Ref(2)P, are both autophagy components that facilitate the targeted selection and sequestration of substrates, which includes protein aggregates (aggrephagy) [1, 18, 23, 29]. As part of its interactions with targeted cargo and other pathway components, the p62 and Ref(2)P proteins are incorporated into new autophagosomes and degraded in the lysosome [18, 26]. Therefore, by taking advantage of this unique feature and by assessing total or insoluble p62/Ref(2)P levels, the relative *in vivo* activity and flux of targeted substrates through the pathway can be partly assessed in cells and whole tissues [30, 31].

Genetic and transgenic studies in *Drosophila* have been used extensively to model the complex *in vivo* cellular processes linked to human aging and neurological disorders. Previously, we have shown that adult flies containing autophagy mutations are short-lived, stress-sensitive and demonstrate a premature build-up of endogenous neural aggregates [5, 18, 25, 32]. Conversely, transgenic expression of the Atg8a protein in fly CNS tissues lowers protein aggregate profiles, preserves behaviors and promotes adult longevity [1, 5, 18]. Therefore, these findings indicate that the common practice of expressing tagged versions of the Atg8a or MAP-LC3 proteins to assess new autophagosome (Atg8a positive punctae) formation and pathway activity may actually alter the long-term *in vivo* profiles of the pathway [30, 33, 34]. Thus, *in vivo* studies focused on aging may require direct characterization of endogenous pathway components to better understand autophagic responses in complex tissues. Examining these dynamic processes in fly and mammalian nervous systems has remained a significant challenge [6, 34–39]. We have recently published on the response of the autophagy pathway occurring in the adult CNS following exposure to traumatic brain injury [24, 40, 41]. During these studies we determined there are dynamic changes in the pathway function that can be detected using confocal imaging and Western blot analysis [40].

In this report, we demonstrate that the majority of Atg8a-positive vesicles, or autophagosomes, within the fly brain are primarily detected in the soma and projections of mature neurons. Further, our detection methodologies are expanded to assess the acute induction of the autophagy pathway in the adult fly nervous system following a fast. From these studies, we identify dynamic age, genetic and gender-specific functional alterations to autophagy. In addition, by utilizing a novel intermittent fasting (**IF**) protocol, we demonstrate IF-treatment also improves the long-term responses of the autophagy pathway following a fast, as well as promotes longevity and maintains behaviors.

## Material and Methods

### Fly stocks, culturing conditions, weight-loss and IF treatment protocol

Canton-S (CS), *w*^*1118*^, *Atg8a*^*1*^, and *Atg8a*^*2*^ flies have been previously described and were obtained from the Bloomington Stock center (Bloomington, IN, USA) [5, 18]. The *chico*^*1*^/Cy stock was a gift from Dr. Montminy’s group (Salk Institute) [18, 42, 43]. Wild-type (**WT**) control flies were F1 offspring generated from crosses between CS females and *w*^*1118*^ male flies. The other genotypes used in this study were also F1 individuals produced through crosses with either CS or *w*^*1118*^ fly strains, as previously described [1, 5, 40]. Adult flies were collected within four hours of eclosion, aged in same-sex cohorts (25 flies per vial), and maintained on standard fly media (agar, molasses, yeast, cornmeal, propionic acid, nipagin) and culturing conditions (25°C, 65% humidity, 12-hour light:dark cycle) [1, 18, 40].

To assess changing weight profiles, four gender-specific cohorts containing 25 young flies (1-week) were weighed four times, starting at 9:00 am. Flies were placed in fasting vials (1% agar, wet fast) and each fly cohort re-weighed after 4, 8 or 24 hours of fasting. To measure weight recovery, fasted male and female flies (8 hours) were returned to standard fly media and permitted to recover overnight (re-feeding 16-hours) before being re-weighed at 9:00 am the following morning [44, 45]. The data is presented as weight (mg) per fly. For all IF studies, flies were maintained using standard conditions until 1-week of age before being exposed to either IF or *ad libitum* treatment conditions. IF-treated flies were turned onto fasting vials (1% agar) three times per week from 9:00 am to 5:00 pm (8-hours). IF-treated flies were placed on *ad libitum* conditions for two full days prior to the initiation of behavioral or protein assays [46]. Longevity profiles were established by counting the number of dead flies three times per week [32]. IF-treated flies were exposed to IF conditions for the duration of longevity studies. Average lifespans and SEM values were generated using MS Excel and P-values were calculated using GraphPad software [5, 25].

### Confocal imaging

**Fig 1:**
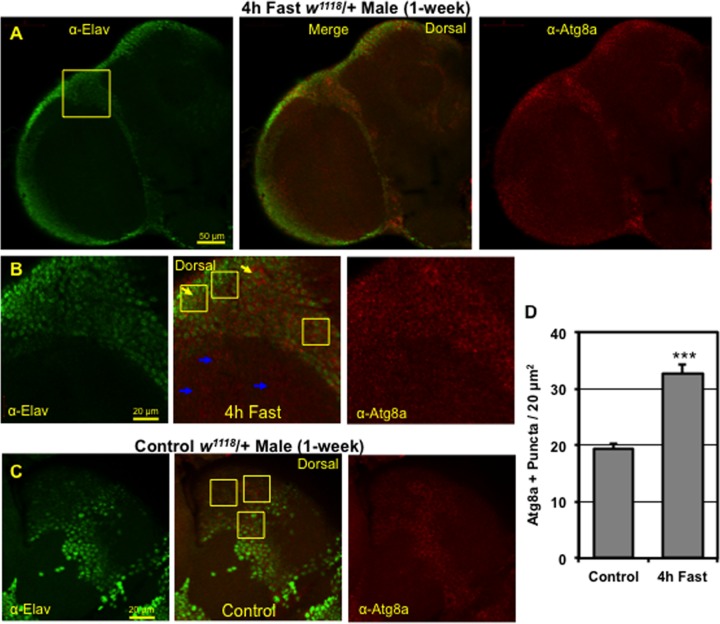
Distribution of Atg8a positive punctae in the adult *Drosophila* CNS. (**A**) Representative confocal image (1.0 μm optical section, top left) of adult male fly brains following a 4-hour fast. Adult brains were co-stained with the anti-Elav neuronal (green) and the anti-Atg8a autophagy (red) markers. (**B**) Higher magnification images (see **Fig 1A** inset) highlight areas enriched with Atg8a positive punctae, which primarily include neuronal soma (cell bodies) and regions of neuropil (blue arrows). Yellow boxes (20 μm^2^) show the location of regions in the CNS that primarily contain neuronal soma that were used to count and establish autophagosome punctae profiles that occur in the adult fly brain. Additional, higher magnification images are included in **S1A and S1B Fig** (regions highlighted by yellow arrows). (**C**) Magnified images from similar brain locations of non-fasted adult male flies stained with anti-Elav and anti-Atg8a antibodies. Yellow boxes indicate regions containing neuronal soma (green) that were used to count Atg8a positive punctae (red). (**D**) Average number of Atg8a positive punctae or autophagosomes in control (n = 53 fields) neural tissues and following a brief 4-hour fast (n = 66 fields). P*** ≤ 0.001.

The heads from young male and female WT flies (*w*^*1118*^/+, 1-week) were collected and the surrounding cuticle and tracheal tissues were dissected away from the adult CNS. Brains were fixed on ice for 45 min (4% paraformaldehyde, 1x PBS), washed in PBST (0.1% Triton X-100 in 1x PBS) and blocked in 5% normal goat serum (5% NGS) [32, 40]. Brains were incubated overnight (4°C) in anti-Elav (1:500 dilution, 9F8A9, Developmental Studies Hybridoma Bank [DSHB], Iowa City, IA, USA) [40, 47] or anti-Repo (1:400 dilution, 8D12, DSHB) [40, 48] antibodies and co-stained with an antibody specific for the fly Atg8a protein (1:250 dilution, E1J4E, human GABARAP, Cell Signaling Technology [CST], Danvers, MA, USA) [40]. Brains were washed with PBST, blocked in 5% NGS and incubated for 3 hours at room temperature with Alexa Fluor-488 (1:250 anti-mouse) or Cy3 (1:250 anti-rabbit) secondary antibodies (Jackson ImmunoReseach Labs, Inc., West Grove, PA, USA) as previously described [1, 18, 40]. Autophagosome counts were obtained from the brains (n = 4) of 1-week old male flies that were maintained on *ad libitum* conditions or fasted for 4-hours. Images from individual confocal Z series (1.5 μm optical section) were taken of the dorsal cortical regions of the adult CNS. The Atg8a-positive punctae were counted in multiple 20 μm^2^ area image fields (see **Fig 1B and 1C**) from flies on *ad libitum* or fasted conditions (4 to 8 hours) [40].

### Antibodies and protein analysis

For protein aggregate analysis, male flies from different ages, genotypes and treatment conditions were collected, flash frozen, and stored at -80°C. Adult head tissues were homogenized using a Bead Ruptor-24 System (Omni International, Kennesaw, GA, USA) for analysis of total proteins or for sequential detergent extraction in Triton X-100 (1.0%) and SDS (2.0%) buffers as described [1, 5, 32]. Protein concentrations for each sample were determined using the DC Protein assay (Bio-Rad, Hercules, CA, USA). For Western blots, 20 μg of protein was loaded per lane and resolved on a 12% Midi-Bis-Tris gel (Bio-Rad), followed by electro-blotting onto Immobilon-P membranes (Millipore Corp., Billerica, MA, USA) using the Trans Blot Turbo system (Bio-Rad). Blots were sequentially probed using anti-Atg8a (E1J4E, CST) [40], anti-Ref(2)P [1], anti-Ubiquitin (P4D1, CST) and anti-Actin (13E5, CST) antibodies at a 1:1,000 dilution [1, 18]. Blots were developed using Thermo Scientific West Dura Substrate (Thermo Scientific Pierce, Rockford, IL, USA) and the ChemiDoc digital Imaging System and Quantity One software (Bio-Rad). ImageJ software (imagej.nih.gov) was used to quantify relative intensity of different proteins, which are also normalized to Actin levels. For each condition a minimum of three replicate samples were used for Western blot analyses.

### Quantitative PCR

Male flies (1-week) were fasted for 0, 4 or 8-hours, collected at each time-point, flash frozen and stored at -80°C. Replicate RNA samples were isolated from 25 heads using Trizol (Thermo Fisher Scientific) and cDNA libraries were generated using the RevertAid First Strand cDNA Synthesis kit, with a combination of random hexamer and oligo-dT primers (Thermo Fisher Scientific). Quantitative PCR was performed on a CFX Connect Real-Time PCR Detection System (Bio-Rad) and Universal PCR SYBR Mix reagents (Bio-Rad). Primer sequences for the *Drosophila Atg8a* gene are available upon request. Melt curve analyses of all qPCR products confirmed the production of a single DNA duplex. The Pfaffl method was used to quantitate expression profiles and *Cyp1* used as a reference gene [1, 5, 40]. Relative mRNA levels of non-fasted control flies were set at 1.0 and subsequent expression levels from different time points were expressed as normalized values.

### Geotaxis response

The *Drosophila* negative geotaxis response protocol and apparatus used in this study has been previously described [1]. Fly cohorts (15–25) were tapped down and digital images of climbing flies were recorded after 5-sec (*w*^*1118*^/+, *Atg8a*^*1*^) or 3-sec (*chico*^*1*^/+) using a Nikon Coolpix L18 camera. Flies were allowed to rest for 1-min between four replicate runs [1, 49]. Digital images were scored for the distance traveled within 5 or 3 seconds (bottom = 0 to top = 6). Replicate runs from each fly cohort were taken at weekly intervals and used to establish age and genotype-specific climbing indexes [1].

### Statistical analysis

Statistical analyses between groups were performed using either Microsoft Excel or GraphPad software and Student’s t-tests (two-tailed, unpaired) were performed to establish P-values. All values are reported as means ± SEM [1].

## Results

### Assessing autophagy profiles in adult *Drosophila* neural tissues

For an initial assessment of autophagic profiles, we examined the distribution of Atg8a-positive punctae or organelles (autophagosomes) in CNS tissues from adult flies fasted for 4 hours (**Fig 1**) [40, 50, 51]. Fasting typically activates the pathway in most tissues, leading to a rapid increase in the number of autophagosomes [30, 34, 52]. However, there have been limited *in vivo* analyses of autophagy profiles and responses under different physiological conditions in neuronal tissues, especially in *Drosophila* [40, 53–55]. Atg8a (red) staining profiles were especially enriched in cortical areas that were positive for the Elav protein, a neuronal soma marker (green, **Fig 1A**) [47]. At higher magnifications Atg8a-positive punctae (yellow arrows) were detected in neuronal cell bodies (soma, also see **S1A and S1B Fig**), as well as in regions that primarily consisted of *Drosophila* neuropil or axonal tracts (blue arrows, **Fig 1B** and **S1C Fig**). In contrast, relatively few glial cells (Repo-positive) contained Atg8a-positive punctae (yellow arrow, **S1D Fig**) [48]. Non-fasted control flies demonstrated similar staining patterns in the CNS cortex regions, albeit at lower levels than fasted flies (yellow arrows, **Fig 1C**) [30, 40, 52]. Quantification of Atg8a-positive autophagosomes showed that following a brief 4-hour fast there was a rapid increase in vesicle numbers (68%) in adult male neural tissues (**Fig 1D**) [35, 53–55]. Therefore, confocal imaging studies indicate that *Drosophila* neural tissues have high levels of basal autophagy that can be further activated by fasting [35, 36].

**Fig 2:**
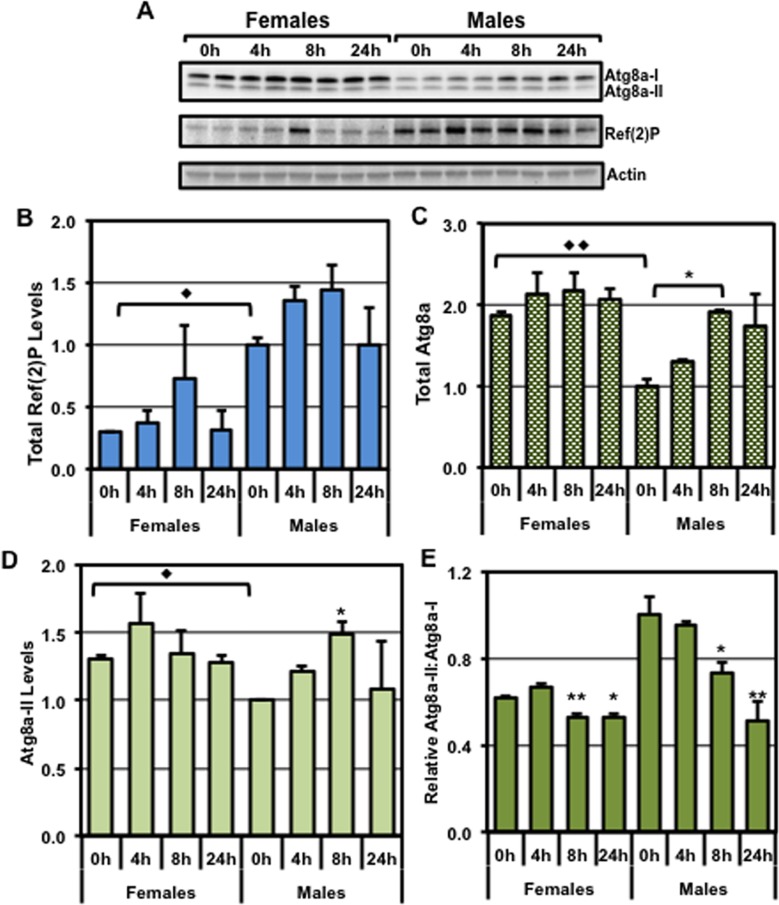
Influence of gender and fasting on autophagic responses and longevity profiles. Young WT female and male flies (*w*^*1118*^*/+*, 1-week) were subjected to 0, 4, 8 or 24 hours of fasting (1% agar). (**A**) Total protein extracts from adult heads were prepared for each condition (n = 3) and used to generate Western blots that were sequentially probed for the Atg8a, Ref(2)P, and Actin proteins. Quantification of the (**B**) Ref(2)P and (**C**) total Atg8a (I+II), and (**D**) Atg8a-II values normalized to Actin loading controls. (**E**) The relative ratio between the Atg8a-II and Atg8a-I proteins. (◆) represents significant differences between genders and (*) represents a difference within a gender specific group. *^,^ ◆P≤ 0.05, **^,^ ◆◆P ≤ 0.01, ***P ≤ 0.001.

To further assess the impact of fasting on autophagic profiles, total head protein extracts from young (1-week) female and male flies were examined for Atg8a and Ref(2)P proteins (**Fig 2**) [40]. Under basal conditions (0-hour fast), there were distinct gender-specific differences in basal autophagic profiles (**Fig 2A**). Female flies showed significantly lower Ref(2)P levels than their age-matched male counterparts (**Fig 2A and 2B**), indicating they have higher basal levels of autophagy in neuronal tissues [56–60]. Coincidently, female flies also exhibited higher Atg8a-II and total Atg8a (I+II) levels (**Fig 2A, 2C and 2D**) [30, 34, 36, 59]. Brief periods of fasting (on 1% agar) did not significantly alter the neural Ref(2)P profiles in female or male flies (**Fig 2A and 2B**). This indicates that changes to total Ref(2)P levels do not necessarily reflect the acute induction of the pathway and in this context would be a more appropriate marker to assess basal autophagy levels. However, male flies that were fasted for 8 hours had a significant increase in both Atg8a-II and total (I+II) profiles (**Fig 2C and 2D**). These protein changes were independent of alterations in *Atg8a* mRNA expression (**S2 Fig**), indicating that the alterations to this key autophagic marker most likely reflect an acute change at the protein level. Female flies, on the other hand, demonstrated more modest changes to these autophagy markers (**Fig 2A–2D**). Interestingly, the parameter that showed the most consistent fasting-induced change for both genders was the reduction in the Atg8a-II:Atg8a-I ratios (**Fig 2E**). Combined, these data suggests that the fasting induced activation of neuronal autophagy can be assessed by the rapid decrease in Atg8a-II:Atg8a-I ratios, regardless of whether an individual group of flies started with higher (female) or lower (male) basal pathway levels [1, 56–59].

### Longevity and autophagic changes associated with IF treatment

Dietary modifications, such as time-restricted feeding and caloric restriction, have been used to modify aging and longevity profiles in various model systems [30, 34, 44, 61–65]. Recently, select intermittent fasting (**IF**) protocols have been shown to positively impact the physiology and metabolic profiles of aging humans and model organisms [61, 65–68]. Since fasted flies demonstrated rapid activation of neuronal autophagy (**Figs 1 and 2**), it suggested that a potential benefit associated with IF-treatment could involve the periodic induction of the pathway [45, 62, 66–70]. However, the long-term impact of IF on the health and neural function of adult *Drosophila* has remained unclear. Previous studies have shown that exposing female flies to short daily fasts (4 or 6-hours) had a negligible effect on longevity profiles [44]. In contrast, flies exposed to a daily time-restricted feeding protocol (12-hour daily fast) maintained more youthful circadian-based behaviors [45, 61, 68, 71].

Therefore, to establish an optimal *Drosophila* intermittent-fasting protocol, male and female flies (1-week) were fasted and weighed at various time points [45, 72]. Flies from both genders exhibited significant weight loss after only 4-hours of fasting and by 24-hours had lost on average ~10% of their total body mass (**S3A and S3B Fig** and **S1A and S1B Table**) [45, 61, 65]. In rodent models, beneficial IF treatments typically involve a modest weight loss, followed by rapid weight recovery once food is reintroduced [45, 61, 65]. Flies that were allowed to recover on *ad libitum* conditions for 16-hours, following an 8-hour fast, quickly regained their original weight (**S3A and S3B Fig** and **S1 Table**). Previous human and rodent studies have also demonstrated that daily fasts are not essential to have a beneficial impact on metabolic rates and neural function [61, 65, 68, 70, 71, 73, 74]. Based on the rapid formation of autophagosomes (**Fig 1D**) and the reduction of Atg8a-II:Atg8a-I ratios following a fast (**Fig 2E**), an IF treatment regimen that exposed young adult flies (1-week) to three weekly, 8-hour fasts was established.

**Fig 3:**
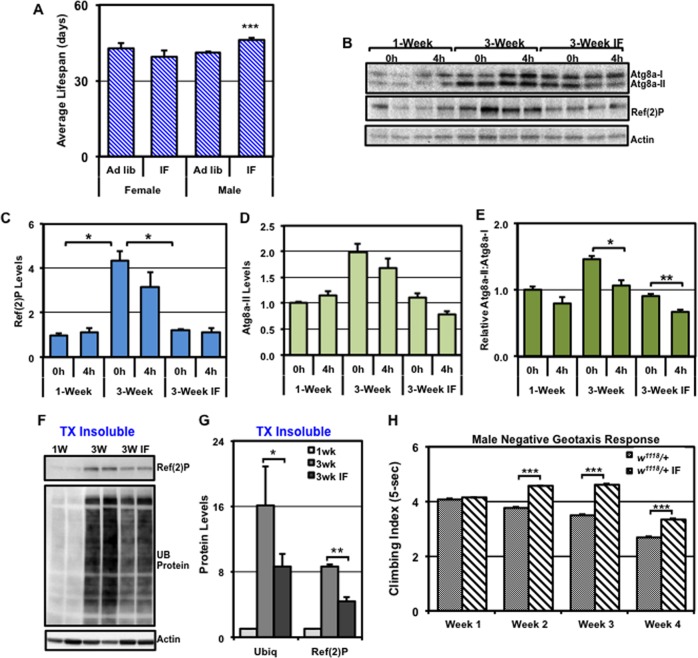
The impact of aging and IF treatment on longevity, neural autophagy, aggregates and behaviors. WT flies (*w*^*1118*^/+) were maintained using standard *ad libitum* or IF treatment conditions beginning at 1-week of age. (**A**) The average lifespan profiles obtained from WT female and male flies. (**B**) Western blots of total protein extracts from control (0h) or fasted (4h) male fly heads at 1-week, 3-week or 3-weeks IF-treated of age (25 per condition, n = 3), were probed for Atg8a, Ref(2)P, and Actin proteins. Quantification of (**C**) Ref(2)P and (**D**) Atg8a-II that were normalized using Actin. (**E**) The relative ratio of the Atg8a-II to Atg8a-I proteins. (**F**) Triton X-100 insoluble protein extracts were prepared from WT male fly at 1-week, 3-week or IF-treated 3-weeks and Western blots probed for Ref(2)P, ubiquitin (UB), and Actin proteins. (**G**) Quantification of Ref(2)P and UB-proteins, normalized using Actin. (**H**) WT (*w*^*1118*^/+) male flies were maintained on *ad libitum* or IF conditions and allowed 2 days of recovery on standard food before the initiation of the NGR assay. The climbing indexes (5-sec) of freely responding *ad libitum* or IF treated flies were performed at weekly intervals starting at 1-week and continuing until 4-weeks of age. *P ≤ 0.05, **P ≤ 0.01, ***P ≤ 0.001.

We examined the impact that IF-treatment has on adult longevity profiles by maintaining 1-week old WT female and male flies (*w*^*1118*^*/+*) on standard *ad libitum* or IF-treatment conditions (three weekly, 8-hour fasts) throughout their entire lifespan. Female longevity was largely unaffected by IF-treatment, while male flies showed a significant increase in average lifespan (12%, **Fig 3A** and **S2 Table**) [44]. The ability of IF-treatment to promote male longevity may reflect the sex-specific differences that acute fasting has on autophagic responses in neural tissues (**Fig 2A–2E**) [44]. Of note, the weights of IF-treated middle-aged (4-weeks) male flies were not significantly different from age-matched *ad libitum* controls (**S1C Table**). Therefore, the modest IF protocol appears to be well tolerated by adult flies and, unlike caloric restriction, does not cause flies to become underweight [44, 45, 61, 64].

To determine whether IF-treatment could also alter long-term autophagic profiles in the aging CNS, we assessed changes in Atg8a and Ref(2)P profiles from neural tissues of young (1-week) and 3-week old *ad libitum* and IF-treated WT male fly cohorts (basal profiles). Non-fasted (0h), *ad libitum* treated middle-aged males (3-weeks) showed an age-dependent build-up of total Ref(2)P (**Fig 3B–3C**) [1, 18, 32]. In contrast, IF-treatment blunted the build-up of Ref(2)P under basal conditions (0h), strongly suggesting that older IF-treated flies maintained more youthful basal autophagy profiles (**Fig 3C**).

Following a fast, Atg8a-II levels did not significantly change in the 3-week old *ad libitum* flies or the age-matched IF-treated flies (**Fig 3D**). In contrast, both 3-week old *ad libitum* and IF-treated flies exhibited lower Atg8a-II:Atg8a-I ratios in the head lysates following a 4h fast (**Fig 3E**), again suggesting this Atg8a-based parameter could be used to assess pathway induction due to fasting [30, 40]. Consistent with improved basal autophagy profiles, IF-treated flies also had a significant reduction in the build-up of Triton X-100 insoluble Ref(2)P and UB-proteins, which naturally accumulates in the neural tissues of middle-aged *Drosophila* (**Fig 3F and 3G**) [1, 5, 18]. Combined, these findings indicate that older flies exposed to a modest IF-treatment schedule maintained more youthful autophagy profiles and potentially neural-based capacities.

### Behavioral changes associated with intermittent fasting

Since IF treatment improved the molecular markers associated with neural aging, we also examined whether IF-treatment could slow the decline of behaviors in aging *Drosophila* [1, 44, 45]. Innate adult fly behaviors are routinely used to examine genetic and age-related changes to neuronal responses [8, 49, 69, 75]. To assess the impact of IF treatment, we examined the negative geotaxis response (**NGR**) of male flies using a customized rapid iterative negative geotaxis (**RING**) assay system [1, 16, 49, 75, 76]. The fly NGR is associated with a robust climbing behavior, which can be influenced by environmental manipulations such as diet and exercise training [16, 75]. In addition, the NGR behavior starts to degenerate at a time (3 to 4-weeks) that coincides with the natural accumulation of neural aggregates [1, 5, 18]. Consistent with previous studies, *ad libitum* flies showed a rapid decrease in climbing abilities starting at 2-weeks, which becomes pronounced by 4-weeks of age (**Fig 3H**) [1, 49]. In contrast, IF-treated males maintained more youthful climbing profiles and continued to outperform control flies for up to 4-weeks (**Fig 3H**) [1].

### The impact of genetics on neuronal aging and autophagy responses

**Fig 4:**
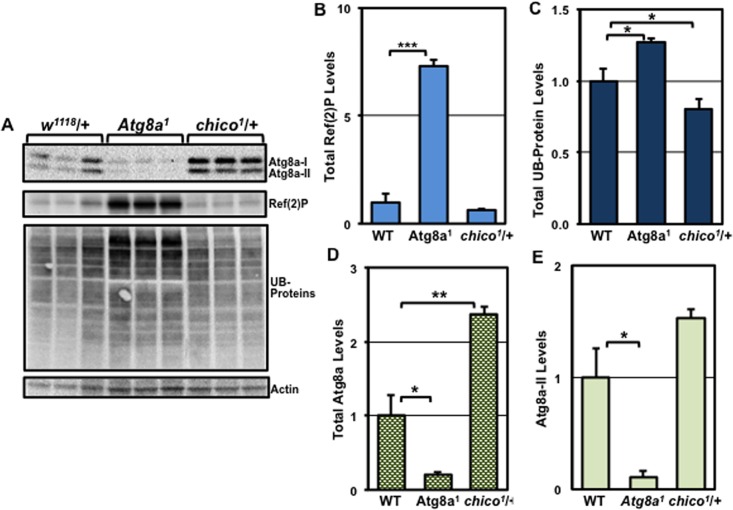
The influence of genetics on basal autophagy profiles occurring in the fly CNS. Age-matched WT (*w*^*1118*^/+), *Atg8a*^*1*^ and *chico*^*1*^/+ male flies (1-week) were collected and heads used to prepare total protein extracts. (**A**) Western blots were probed for Atg8a, Ref(2)P, ubiquitin (UB), and Actin proteins (n = 3). Quantification of total (**B**) Ref(2)P, (**C**) UB-proteins, (**D**) total Atg8a (I+II), and (**E**) Atg8a-II proteins, normalized using Actin. *P ≤ 0.05, **P ≤ 0.01, ***P ≤ 0.001.

Our studies have demonstrated that age- and gender-induced alterations to neuronal autophagy profiles can be quantified by Western analysis of key pathway markers (**Figs 2 and 3**). To determine if this technique could also be used to detect genetic-based differences to pathway responses, we assessed the neural profiles of young *Atg8a*^*1*^ (autophagy-impaired) [5, 18] and heterozygous *chico*^*1*^/+ (autophagy-enhanced) male flies [18, 43, 77] Previous studies have demonstrated these fly genotypes produce normal appearing adults that have divergent lifespan and neural aggregate profiles [5, 18, 25, 43]. Indeed, young males (1-week) from each genotype showed unique protein patterns that reflect the endogenous basal autophagic profiles in the nervous system (**Fig 4A–4E**). When compared to WT males, young *Atg8a*^*1*^ mutants exhibited a robust accumulation of total Ref(2)P indicating diminished basal autophagy profiles in these flies, as well as a buildup of UB-proteins (**Fig 4A–4C**). As expected, *Atg8a*^*1*^ mutants also showed reduced Atg8a protein levels (**Fig 4A and 4D and 4E**). In contrast, *chico*^*1*^/+ flies exhibited lower UB-protein profiles and Ref(2)P levels also trended lower, further suggesting this genotype starts with elevated pathway activity or capacity (**Fig 4A–4C**). In addition, under basal conditions the *chico*^*1*^/+ males had elevated total Atg8a and Atg8a-II levels (**Fig 4A and 4D and 4E**). Combined, these results demonstrate that additional considerations should be made when comparing different fly genotypes with regards to their basal autophagy profiles.

### Neuronal responses of flies with insulin signaling defects (*chico*^*1*^*/+*)

**Fig 5:**
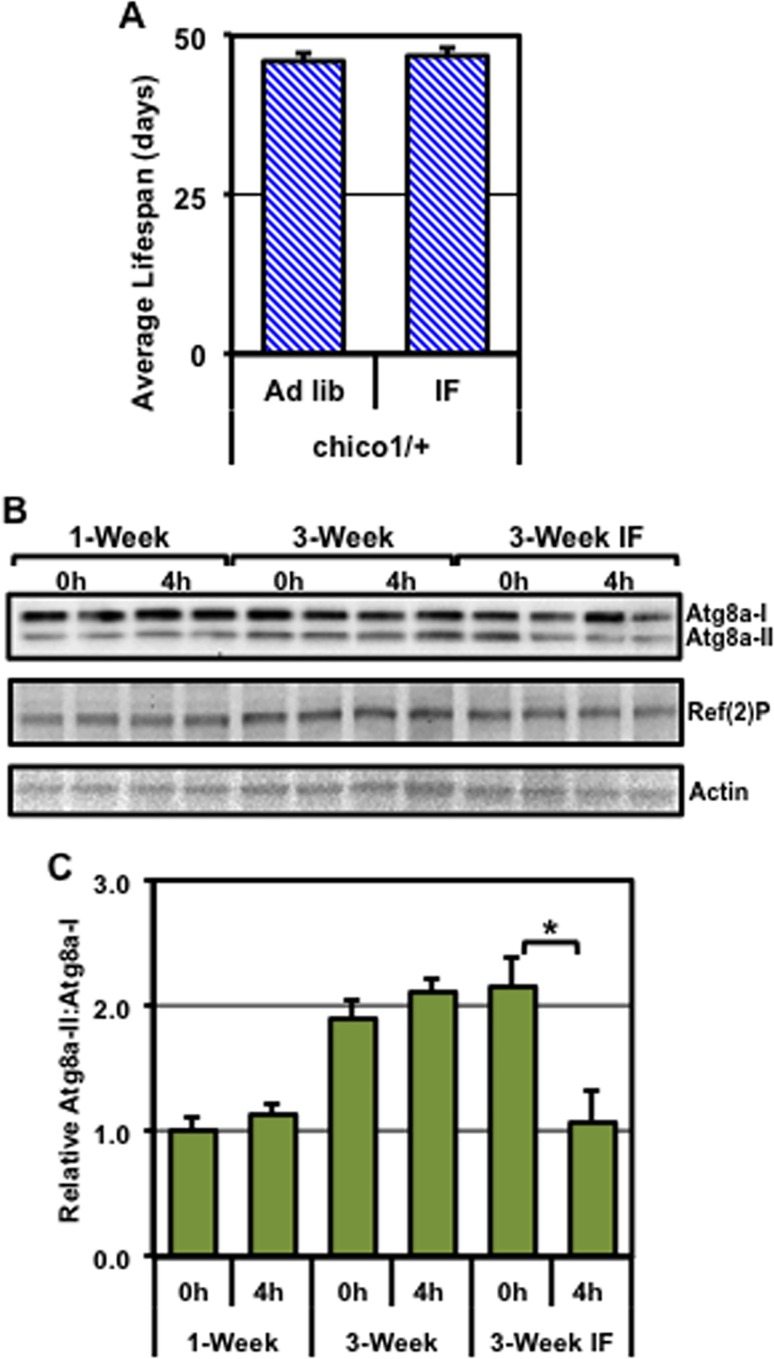
Changes to the lifespan and autophagy profiles of *chico*^*1*^/+ male flies. (**A**) The average lifespan profiles of *chico*^*1*^/+ mutant male flies exposed to *ad libitum* or IF treatment conditions, starting at 1-week of age. (**B**) Total head protein extracts from control (0h) or fasted (4h) male flies at 1-week, 3-week or IF-treated 3-week of age (n = 3), were used for Western blot analysis of the Atg8a, Ref(2)P, and Actin proteins. (**C**) The relative ratio of Atg8a-II to Atg8a-I proteins. *P ≤ 0.05.

Given the positive effects that IF treatment had on the lifespan and autophagic induction in WT flies, we tested whether our protocol could benefit flies that have enhanced basal autophagy. Unlike WT flies, IF-treatment did not improve the average lifespan profiles of male *chico*^*1*^/+ flies (**Fig 5A** and **S2 Table**) or improve their NGR (**S4A Fig**). Furthermore, total Ref(2)P levels were largely unchanged in neural samples from young, 3-week *ad libitum*, and 3-week IF-treated *chico*^*1*^/+ fly cohorts (**Fig 5B** and **S4B Fig**). This was consistent with young *chico*^*1*^/+ flies having elevated basal levels of autophagy (**Fig 4**), which was largely maintained in middle-aged adults (**Fig 5**and **S4 Fig**) [42, 43]. Again, total Atg8a and Atg8a-II levels failed to accumulate in young or 3-week old *chico*^*1*^/+ flies following a fast (**S4C and S4D Fig**). Interestingly, IF-treated *chico*^*1*^/+ flies exposed to a 4-hour fast showed a significant reduction in Atg8a-II:Atg8a-I ratios, while young and 3-week old *ad libitum* controls did not (**Fig 5C**). These data demonstrate that, although IF treatment likely promoted the acute fasting-induced activation of autophagy, the overall benefit to *chico*^*1*^/+ flies is modest, which is consistent with the results from other dietary studies for this fly genotype [43].

### Neuronal responses of adult autophagy mutants (*Atg8a*^*1*^)

**Fig 6:**
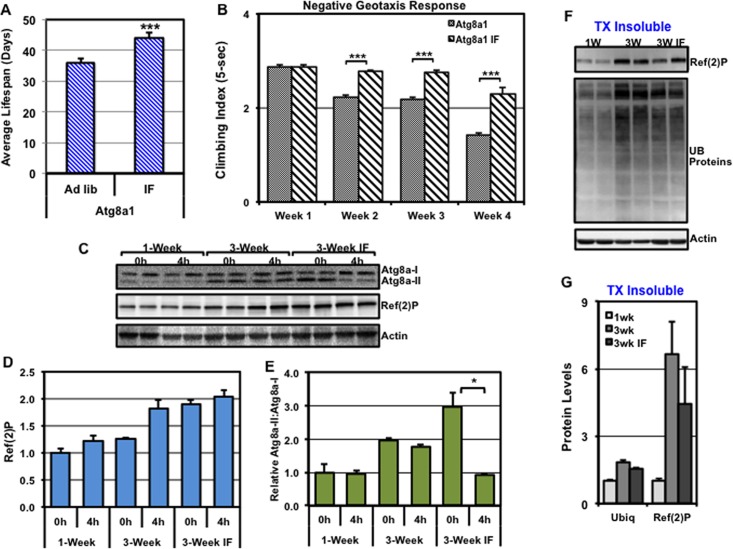
IF-dependent changes to the lifespan and autophagy profiles of *Atg8a*^*1*^ mutant flies. (**A**) The average lifespan profiles of *Atg8a*^*1*^ mutant male flies were exposed to *ad libitum* or IF treatment conditions starting at 1-week of age. (**B**) The climbing indexes (5-sec) of freely responding *ad libitum* or IF treated *Atg8a*^*1*^ male flies, which were performed at weekly intervals starting at 1-week and continuing until 4-weeks of age. (**C**) Western blots containing total protein extracts prepared from control (0h) or fasted (4h) *Atg8a*^*1*^ male fly heads taken at 1-week, 3-week or IF-treated 3-week of age (n = 3) were sequentially probed for Atg8a, Ref(2)P, and Actin proteins. (**D**) Quantification of Ref(2)P protein levels, normalized using Actin. (**E**) The relative ratio of Atg8a-II to Atg8a-I protein levels. (**F**) Western blots of Triton X-100 insoluble head extracts from 1-week, 3-week or IF-treated 3-week old of *Atg8a*^*1*^ male flies that were sequentially probed for the Ref(2)P, ubiquitin (UB), and Actin proteins. (**G**) Quantification of Ref(2)P and UB-proteins in the Triton X-100 insoluble fraction, normalized using Actin. *P ≤ 0.05, **P ≤ 0.01, ***P ≤ 0.001.

To determine whether IF-treatment could improve global physiological profiles of flies that have impaired autophagy, the lifespan profiles of *ad libitum* and IF-treated *Atg8a*^*1*^ were examined [5, 18, 25]. *Atg8a*^*1*^ mutant fly strains exhibited a significant increase in average longevity following IF-treatment, with a 22% increase in median lifespan extension (**Fig 6A** and **S2 Table**). Additional IF studies using a second stronger mutant strain, *Atg8a*^*2*^, resulted in a similar 27% increase in lifespan (**S2 Table**). Correlating with improved lifespans, IF-treated *Atg8a*^*1*^ males also showed more youthful NGR profiles, as compared to age-matched *ad libitum* controls (2 to 4-weeks, **Fig 6B**).

Interestingly, IF-treatment led to a build-up of Ref(2)P in the basal state in total neural extracts from *Atg8a*^*1*^ flies (**Fig 6C and 6D**). However, IF-treated flies showed a fasting-induced decrease in Atg8a-II:Atg8a-I ratios (**Fig 6E**) and both UB-protein and Ref(2)P levels trended lower in the Triton X-100 insoluble fraction (**Fig 6F and 6G**), which was consistent with improved basal and activated pathway profiles [1, 18]. Combined, these data indicate that IF-treatment does not significantly change basal autophagy levels in *Atg8a*^*1*^ mutants, but in aged cohorts may improve pathway activation following a fast.

## Discussion

The systematic analysis of endogenous autophagic responses occurring in the nervous system has been limited, especially in a genetically tractable system such as *Drosophila* [34–36, 39]. Alirezaei et al. demonstrated that GFP-tagged-LC3 expressed in the nervous system of transgenic mice could be used to detect autophagosome formation following a fast [34, 41]. However, our group has previously shown that either transgenic overexpression of endogenous Atg8a or a UAS-GFP-Atg8a construct within the fly CNS can alter pathway dynamics, which is reflected in lower neural aggregate levels, the maintenance of behaviors and enhanced adult longevity [1, 5, 18]. Therefore, using the GFP-Atg8a protein to mark and follow autophagic profiles (autophagosome formation) in aging neural tissues may complicate the assessment of endogenous basal and acute pathway responses. In this study, we utilized a commercially available antibody for imaging and Western based studies to directly detect the fly Atg8a protein and characterize endogenous pathway responses. We identified regions of the adult *Drosophila* CNS, primarily in areas containing neural cell bodies or soma, which were highly enriched in Atg8a-positive punctae (**Fig 1**and **S1 Fig**) [1]. Interestingly, we also observed a significant increase in the number of endogenous autophagosomes (~70%) following even a brief 4h fast, indicating that fly neurons can respond quickly to a fasting stimulus and further induce autophagy (**Fig 1D**).

Fasting is known to activate the autophagy pathway in many tissues and changes in Atg8a-II levels are often used to assess the acute induction of the pathway [30, 34]. However, the assessment and the detection of endogenous Atg8a-II or LC3-II changes in the nervous system has not been routinely employed as a method to detect pathway activation [30]. In young (1-week) female flies, we did not observe a rapid build-up in Atg8a-II levels following 4 or 8 hours of fasting, although male flies showed an increase after an 8-hour fast (**Fig 2D**). In contrast, flies from both genders exhibited a significant reduction in Atg8a-II:Atg8a-I ratios following an 8 or 24 hour fast (**Fig 2E**). Combined, these data further underscore the use of Atg8a-II:Atg8a-I ratios to assess changing pathway profiles, such as in times of fasting, in the adult *Drosophila* neural tissues. As with other Western-based studies, we propose that comparisons of Atg8a-II:Atg8a-I ratios from various treatment conditions must be performed on the same blot. The concern is that slight differences in transfer, hybridization and exposure conditions on different blots could complicate the assessment of dynamic changes in protein ratios. These studies are the first to our knowledge that have directly examined acute changes to the endogenous autophagy pathway within the adult fly CNS.

By assessing Atg8a-II:Atg8a-I ratios, as well as Ref(2)P levels, we demonstrate that gender, aging, dietary changes and genetic differences have an impact on the autophagic responses that occur in *Drosophila* neurons. One of the more unexpected findings from this study was the dramatic gender-based difference in autophagic profiles [56, 57, 60]. Young male flies started with lower basal autophagy levels, but demonstrated a rapid induction of the pathway following fasting (**Fig 2**). Conversely, females started with higher autophagic profiles, but had a more limited fasting response (**Fig 2**). These data highlight that gender should also be taken into consideration when performing detailed analysis of autophagic profiles in flies or other model organisms.

*Drosophila* genetic approaches have been used extensively to study complex *in vivo* autophagic responses, especially those occurring in the larval fat body [29, 30, 52]. For this study the *Atg8a*^*1*^ and *chico*^*1*^*/+* fly strains were selected for the characterization of adult neuronal autophagic responses partly due to these genotypes having established neural aggregate profiles that are consistent with suppressed (*Atg8a*^*1*^) or enhanced (*chico*^*1*^*/+*) autophagy [5, 18, 42, 78]. These genetic backgrounds also produce viable, normal appearing adult flies (body size, fecundity) and have well-defined longevity profiles [5, 42, 77, 78]. In regards to *Atg8a*^*1*^ flies, this hypomorphic *Atg8a* allele results in only modest autophagy pathway impairments; however, these flies still maintain the potential ability to up-regulate the pathway [5, 18]. In this study, we confirmed that young *Atg8a*^*1*^ mutant males have suppressed neuronal autophagy (elevated Ref(2)P protein levels, **Fig 4**) and exhibited reduced NGR profiles that are consistent with modest behavioral impairments (**Fig 6**) [1, 40]. Unlike WT flies, the *Atg8a*^*1*^ mutants (3-weeks) did not exhibit a decrease in Atg8a-II:Atg8a-I ratios following an acute fast (**Fig 6**), which suggests that autophagic responses are further impaired. However, IF treatment led to a fasting-induced reduction in the Atg8a-II:Atg8a-I ratios in *Atg8a*^*1*^ flies indicating improved autophagic responses. Further, IF treatment in aged *Atg8a*^*1*^ mutants maintained NGR profiles (**Fig 6)** and extended the lifespans of both *Atg8a*^*1*^ and *Atg8a*^*2*^ mutant males (**Fig 6**and **S2 Table**). These data demonstrate that flies that start with impaired autophagic responses can exhibit aging-associated benefits following IF treatment (**Fig 6)**. Although, markers of neural health were improved in IF-treated *Atg8a*^*1*^ flies, an unanticipated increase in whole tissue lysate Ref(2)P levels was detected, which is normally used as an *in vivo* marker of impaired autophagic flux [18, 30, 40]. Although not common, there are certain conditions where the build-up of Ref(2)P/p62 does not closely correlate with impaired autophagic flux [30, 79]. Whether the maintenance of Ref(2)P levels is dissociated from the enhanced autophagy observed in IF-treated *Atg8a*^*1*^ flies remains to be determined.

The *chico*^*1*^*/+* flies represent a genotype that has a modest impairment of insulin-signaling, which does not impact growth-development processes or lead to insulin-resistance [42, 43, 77]. Consistent with enhanced neural autophagy, *chico*^*1*^*/+* flies showed reduced Ref(2)P protein levels and had extended lifespans (**Fig 5 and S2 Table**). Similarly, modest insulin-signaling pathway impairment in heterozygous *Irs2* knockout mice (mammalian *chico* homolog) promotes longevity and upregulates the autophagy pathway [80, 81]. Although previous studies have highlighted that the dysregulation of insulin-signaling in obesity or Type-II diabetes is a risk factor for the development of neurological diseases [17, 65, 82], these studies suggest that simply reducing insulin signaling in the adult CNS is not a causative factor for neural decline [80, 81]. In contrast to WT and *Atg8a*^*1*^ flies, *chico*^*1*^*/+* flies were minimally impacted by IF treatment. IF-treated *chico*^*1*^*/+* flies did not display improved behavioral responses or improved longevity and several key autophagic markers were largely unchanged (**Fig 5**, **S4 Fig** and **S2 Table**). This is consistent with previous studies showing dietary modifications, such as caloric restriction, did not extend longevity in these flies [42, 77]. Combined, these data suggest that *chico*^*1*^/+ flies may be at near maximal levels of autophagic capacity, which limits IF-induced autophagy pathway upregulation to extend lifespan further.

Our findings in aged *Drosophila* underscore how environmental factors, such as IF-treatment or other modest dietary manipulations could influence the long-term maintenance of the nervous system. Different IF protocols have been shown to stabilize weight profiles, improve insulin sensitivity and lower markers associated with age-related stress and inflammation [61–64, 73, 82, 83]. The cellular mechanisms that mediate the beneficial effects of IF-based interventions in humans are not yet fully characterized, but activation of autophagy has been implicated in facilitating at least some of the beneficial effects of limited diets [45, 61, 63, 73, 74]. Our studies highlight that IF treatment does improve the long-term *in vivo* autophagic responses in the aging *Drosophila* CNS across diverse genotypes [5, 42, 43]. In summary, our studies highlight novel methods (imaging and Western blot-based) to examine the induction of the autophagy pathway in the adult fly CNS. In addition, IF treatment improved the neuronal autophagic response of *Drosophila*, as well as promoted the long-term maintenance of behaviors and longevity profiles.

## Supporting Information

S1 Table Changing Adult *Drosophila* Weight Profiles. The *N values represent weights of different fly cohorts (25 per conditions) following a fast and re-feeding At least 125 individual flies were used for each study. **A.** Average adult female fly (*w*^*1118*^*/+*) weights (mg), SEM and percentage weight change that occurred following fasting and an overnight re-feeding. **B.** Average adult male fly (*w*^*1118*^*/+*) weights (mg), SEM and the percentage weight change that occurred following fasting and an overnight re-feeding. **C.** The average weights (mg) of WT male flies (*w*^*1118*^*/+*) at 1-week and 4-weeks of age maintained using *ad librium* conditions or after 3-weeks of IF-treatment (4-weeks of age).
(PDF)

S2 Table The longevity profiles of control and IF treated adult *Drosophila*. Statistical description of the average lifespan (days), SEM, N and P values determined between *ad libitum* and IF-treatment gender and genotype fly cohorts. The percentage change in average longevity is also presented.
(PDF)

S1 Fig Cellular and tissue specific distribution of Atg8a positive punctae within the adult *Drosophila* CNS. Confocal images adult fly brains co-stained for autophagic vesicle marker (Atg8a) and neuronal (Elav) or glial (Repo) markers. (**A-B**) Higher magnification images of neural soma (anti-Elav, green) and Atg8a positive punctae (anti-Atg8a, red) taken from CNS regions highlighted from **Fig 1B** (see yellow arrows in **Fig 1B**). (**C**) Representative confocal images of a female fly CNS (8 hour fast) show similar staining patterns to those of male flies seen in **Fig 1**(n = 10 brains). The location of neuronal soma (cell bodies, yellow arrows) and regions primarily consisting of neuropil are indicated (blue arrows). (**D**) Representative confocal images of a male CNS (8h fast) co-stained for glial (anti-Repo, green) and autophagy (anti-Atg8a, red) markers in the adult CNS (n = 15 brains). Magnified images (inset) show a single Repo-positive glial cell that contains Atg8a positive punctae (yellow arrow). Most glial cells showed limited Atg8a staining (yellow circles).
(TIFF)

S2 Fig Fasting induced changes to *Atg8a* mRNA profiles. Triplicate cohorts of young (*w*^*1118*^/+) adult WT male flies were fasted for 0, 4 or 8-hours, flash frozen and total RNA isolated and used for qRT-PCR analysis of *Atg8a* message levels. Values were normalized using *Cyp1* as a reference gene.
(TIF)

S3 Fig Weight Profiles of Fasted Female and Male *Drosophila*. Starting at 9:00 am, triplicate weights of (**A**) female and (**B**) male fly cohorts (*w*^*1118*^/+, 25 flies per group) were obtained following 0, 4, 8 or 24-hour fast (125 total flies). Fly groups fasted for 8h were placed back onto standard media starting at 5:00 pm and allowed to re-feed overnight (16h) before being re-weighed again the following day at 9:00 am (red columns and arrows). *** P ≤ 0.001.
(TIF)

S4 Fig Age and IF-dependent changes to the behavior, autophagic, and protein aggregate profiles of *chico*^*1*^*/+* mutant male flies. Young outcrossed *chico*^*1*^*/+* male flies (1-week) were maintained using *ad libitum* or IF-treatment conditions. (**A**) The climbing indexes (3-sec) of freely responding *ad libitum* or IF-treated *chico*^*1*^*/+* male flies that were performed at weekly intervals starting at 1-week and continuing until 4-weeks of age. (**B**) Quantified Ref(2)P, (**C**) total Atg8a (I+II), and (**D**) Atg8a-II protein profiles protein of *chico*^*1*^*/+* neural samples illustrated in **Fig 5B**, were corrected using Actin as a loading control.
(TIF)

## Abbreviations

Atg: autophagy-specific gene
CNS: central nervous system
IF: intermittent fasting
NGR: negative geotaxis response
Ref(2)P: refractory to sigma P
RING: rapid iterative negative geotaxis
ROS: reactive oxygen species
SQSTM1: Sequestosome-1
IRS: Insulin receptor substrate
TOR: target of rapamycin
UB-proteins: ubiquitinated proteins
WT: wild-type
