# Supplementary material for: Assessing Basal and Acute Autophagic Responses in the Adult Drosophila Nervous System: The Impact of Gender, Genetics and Diet on Endogenous Pathway Profiles
Source: PLoS One. 2016 Oct 6;11(10):e0164239. doi: 10.1371/journal.pone.0164239 (PMC5053599; doi:10.1371/journal.pone.0164239)
Supplement: S1 Table — The *N values represent weights of different fly cohorts (25 per conditions) following a fast and re-feeding At least 125 individual flies were used for each study. A. Average adult female fly (w1118/+) weights (mg), SEM and percentage weight change that occurred following fasting and an overnight re-feeding. B. Average adult male fly (w1118/+) weights (mg), SEM and the percentage weight change that occurred following fasting and an overnight re-feeding. C. The average weights (mg) of WT male flies (w1118/+) at 1-week and 4-weeks of age maintained using ad librium conditions or after 3-weeks of IF-treatment (4-weeks of age). (PDF) [file pone.0164239.s001.pdf]

S1 Table. Changing adult *Drosophila* weight profiles

A. Females	N	Average	SEM	% Change
0h	16*	1.170	0.015	
4h IF	8	1.100	0.003	-0.060
8h IF	8	1.058	0.014	-0.096
24h IF	8	1.030	0.005	-0.120
Re-Feed	12	1.181	0.003	+0.116
**B. Males**	**N**	**Average**	**SEM**	**% Change**
0h	24*	0.787	0.003	
4h IF	12	0.763	0.003	-0.026
8h IF	12	0.751	0.003	-0.046
24h IF	12	0.699	0.008	-0.111
Re-Feed	21	0.777	0.005	-0.012
**C. Aged Males**	**N**	**Average**	**SEM**	
1-week	12*	0.804	0.038	
4-week	12	0.811	0.034 (ns)	
4-week IF	12	0.817	0.028 (ns)	

*N values represent weights flies (25) within multiple cohorts. At least 100 individual flies were used for each study.
