# Supplementary material for: Assessing Basal and Acute Autophagic Responses in the Adult Drosophila Nervous System: The Impact of Gender, Genetics and Diet on Endogenous Pathway Profiles
Source: PLoS One. 2016 Oct 6;11(10):e0164239. doi: 10.1371/journal.pone.0164239 (PMC5053599; doi:10.1371/journal.pone.0164239)
Supplement: S2 Table — Statistical description of the average lifespan (days), SEM, N and P values determined between ad libitum and IF-treatment gender and genotype fly cohorts. The percentage change in average longevity is also presented. (PDF) [file pone.0164239.s002.pdf]

S2 Table. Longevity profiles

Genotype	Average (days)	SEM	N	P Value	% Change
***w*^{1118}/+ males**	41	0.6	322		
***w*^{1118}/+ IF males**	46	1.1	217	<0.0001*	↑ 12.2
***chico*^{1}/+ males**	55.8	1.1	121		
***chico*^{1}/+ IF males**	57	1.1	120	0.438*	↑ 1.1
***Atg8a*^{1} males**	36	1.4	127		
***Atg8a*^{1} IF males**	44	1.7	104	0.0003*	↑ 22.3
***Atg8a*^{2} males**	22.5	1.3	83		
***Atg8a*^{2} IF males**	28.6	1.0	143	0.0002*	↑ 27.1

* P values determined between *ad libitum* and IF-treatment gender and genotype fly cohorts.
