# Supplementary figures and images for: Assessing Basal and Acute Autophagic Responses in the Adult Drosophila Nervous System: The Impact of Gender, Genetics and Diet on Endogenous Pathway Profiles
Source: PLoS One. 2016 Oct 6;11(10):e0164239. doi: 10.1371/journal.pone.0164239 (PMC5053599; doi:10.1371/journal.pone.0164239)

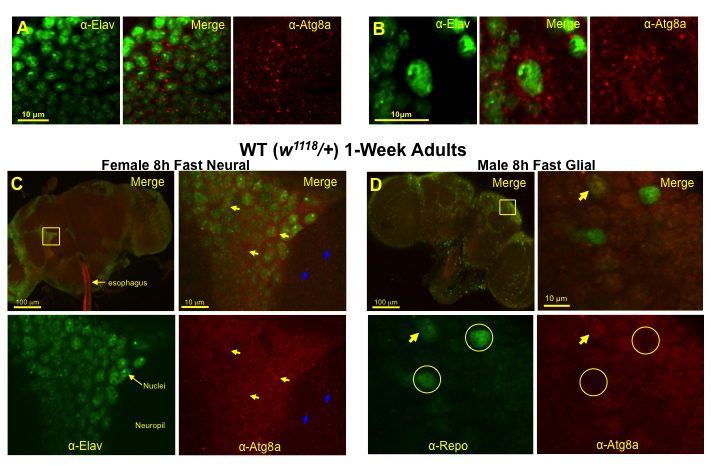

Supplement: S1 Fig — Confocal images adult fly brains co-stained for autophagic vesicle marker (Atg8a) and neuronal (Elav) or glial (Repo) markers. (A-B) Higher magnification images of neural soma (anti-Elav, green) and Atg8a positive punctae (anti-Atg8a, red) taken from CNS regions highlighted from Fig 1B (see yellow arrows in Fig 1B). (C) Representative confocal images of a female fly CNS (8 hour fast) show similar staining patterns to those of male flies seen in Fig 1(n = 10 brains). The location of neuronal soma (cell bodies, yellow arrows) and regions primarily consisting of neuropil are indicated (blue arrows). (D) Representative confocal images of a male CNS (8h fast) co-stained for glial (anti-Repo, green) and autophagy (anti-Atg8a, red) markers in the adult CNS (n = 15 brains). Magnified images (inset) show a single Repo-positive glial cell that contains Atg8a positive punctae (yellow arrow). Most glial cells showed limited Atg8a staining (yellow circles). (TIFF) [file pone.0164239.s003.tiff]

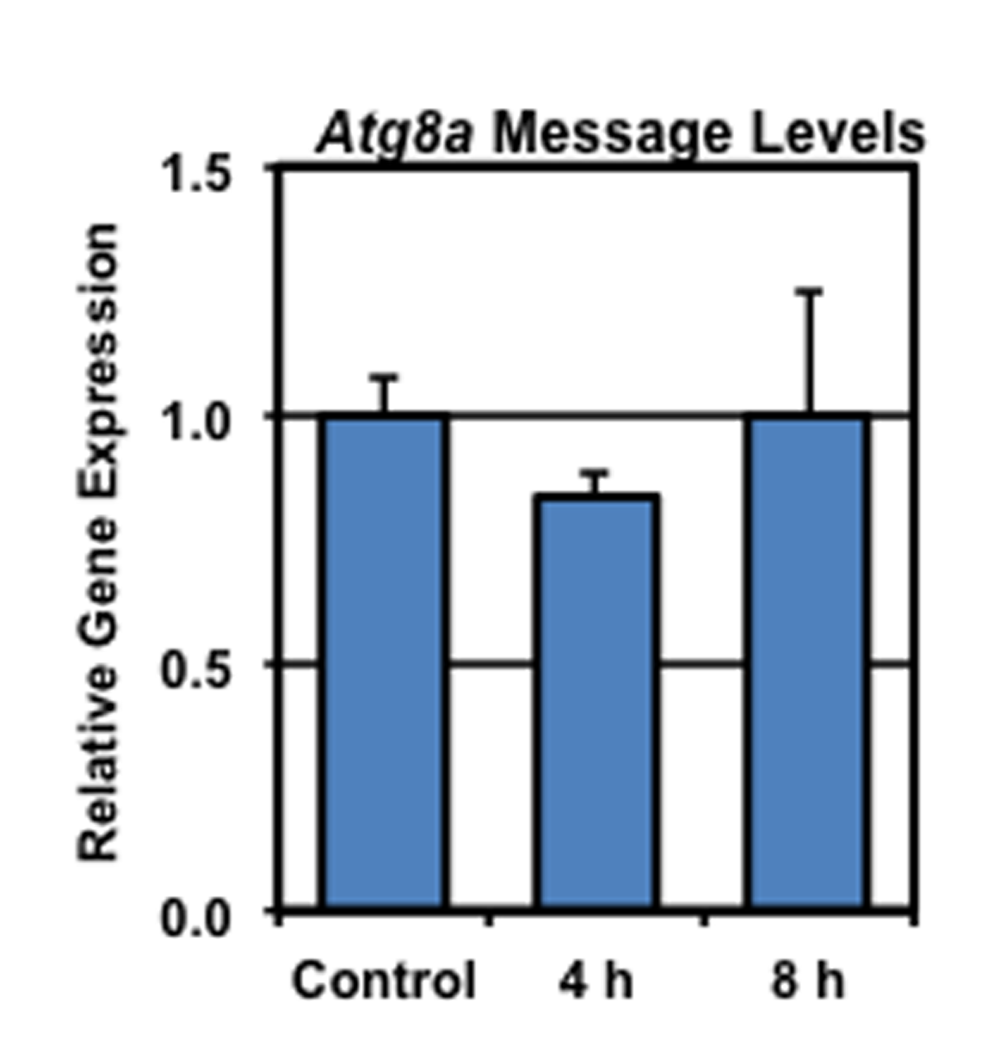

Supplement: S2 Fig — Triplicate cohorts of young (w1118/+) adult WT male flies were fasted for 0, 4 or 8-hours, flash frozen and total RNA isolated and used for qRT-PCR analysis of Atg8a message levels. Values were normalized using Cyp1 as a reference gene. (TIF) [file pone.0164239.s004.tif]

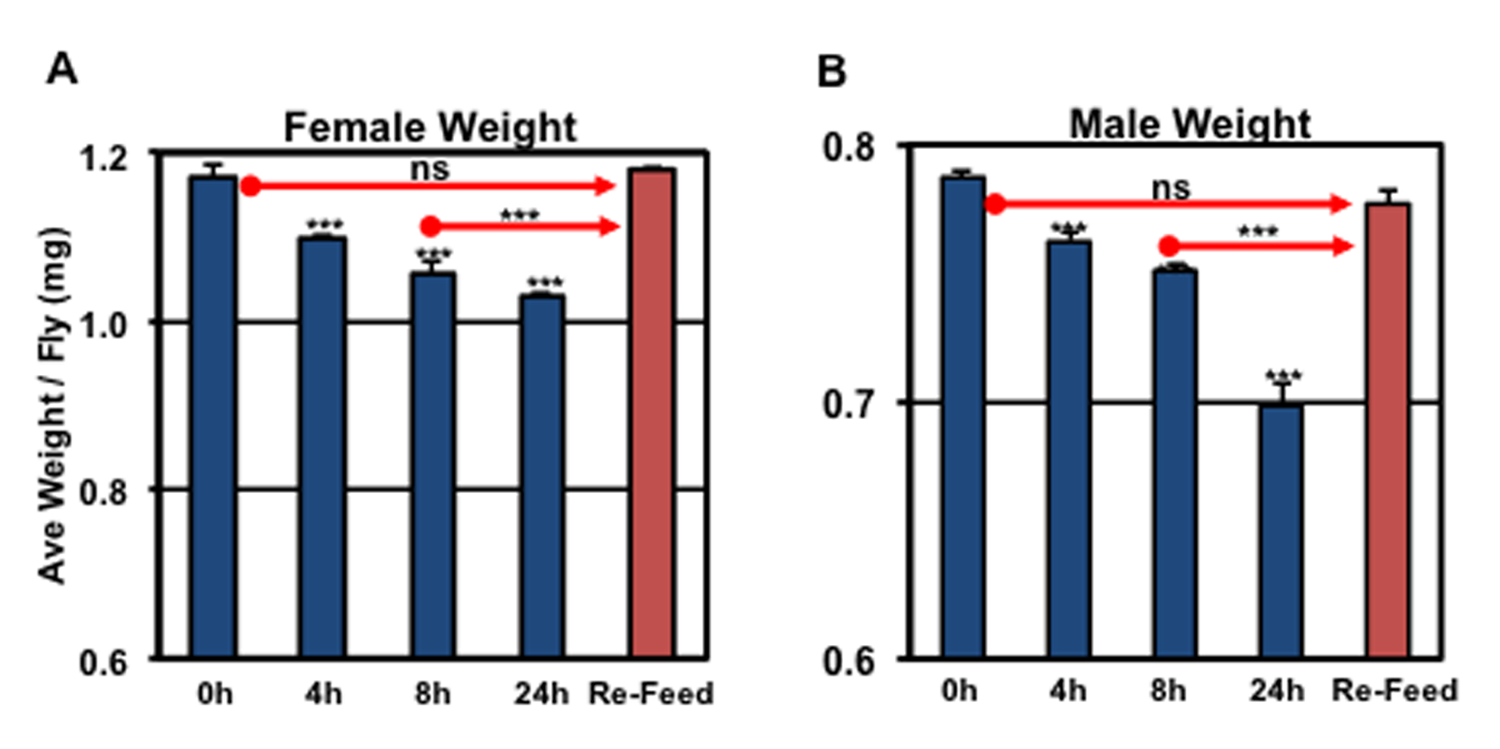

Supplement: S3 Fig — Starting at 9:00 am, triplicate weights of (A) female and (B) male fly cohorts (w1118/+, 25 flies per group) were obtained following 0, 4, 8 or 24-hour fast (125 total flies). Fly groups fasted for 8h were placed back onto standard media starting at 5:00 pm and allowed to re-feed overnight (16h) before being re-weighed again the following day at 9:00 am (red columns and arrows). *** P ≤ 0.001. (TIF) [file pone.0164239.s005.tif]

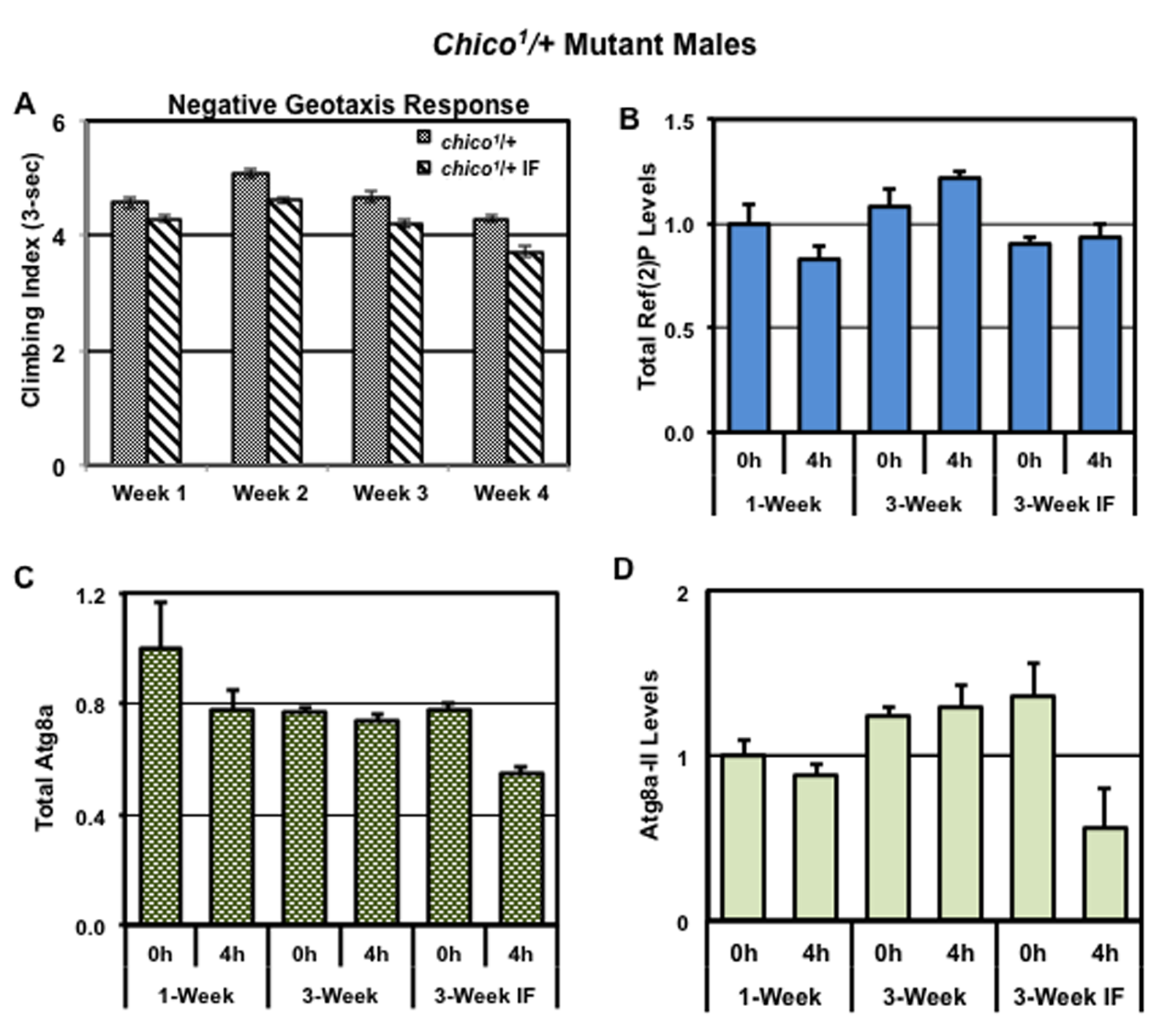

Supplement: S4 Fig — Young outcrossed chico1/+ male flies (1-week) were maintained using ad libitum or IF-treatment conditions. (A) The climbing indexes (3-sec) of freely responding ad libitum or IF-treated chico1/+ male flies that were performed at weekly intervals starting at 1-week and continuing until 4-weeks of age. (B) Quantified Ref(2)P, (C) total Atg8a (I+II), and (D) Atg8a-II protein profiles protein of chico1/+ neural samples illustrated in Fig 5B, were corrected using Actin as a loading control. (TIF) [file pone.0164239.s006.tif]
